# Educational intervention for the main caregiver of primiparous women to promote breastfeeding and the association between prolactin and nutritional parameters

**DOI:** 10.7189/jogh.13.04046

**Published:** 2023-04-21

**Authors:** Eva Pilar López, Sergio González, Mercedes Sánchez

**Affiliations:** 1 Doctoral School in translational Medicine San Pablo CEU University, Madrid, Spain; 2 Department of Nursing, Santa Teresa de Jesus, Catholic University, Avila, Spain; 3 Department of Preventive Medicine and Public Health, Santa Teresa de Jesus, Catholic University, Avila, Spain

## Abstract

**Background:**

In the last two years, breastfeeding rates have experienced a notable decline worldwide. Only 46% of women breastfeed their children, the figure being much lower in primiparous women. Breastfed milk is the ideal food for babies; its benefits for the health of mothers and babies are scientifically proven. Several studies show that babies who are not breastfed have a higher risk of getting sick. This fact gives rise to an important public health problem. The aim of this paper is to describe the association between presence of the caregiver in health education and increasing rates of breastfeeding.

**Methods:**

We conducted an observational study (cohort) in a population of primiparous pregnant women (n = 88), and their main caregivers belonging to a region of central Spain. The development, content and implementation of the intervention consisted of: 1) obtaining the blood levels of pregnant women (prolactin, folic acid, vitamin B12 and transferrin) before health education (13-26 weeks of pregnancy), 2) carry out health education with two groups: A (44 pregnant women with caregivers) and B (44 without caregivers), 3) obtain the same blood levels as in the first intervention, 15 days after delivery, and finally the evaluation of the intervention with breastfeeding rates.

**Results:**

The levels of prolactin (288.57 ± 107.46 nanogrammes per millilitre (ng / ml)), folic acid (16.93 ± 4.09 ng / ml), vitamin B12 (505.05 ± 213.97 picogrammes (pg) / ml) and transferrin (296.82 ± 67.61 milligrammmes per decilitre (mg / dl)) were higher in pregnant women who attended the health education program with a caregiver than in pregnant women who attended alone: prolcoactin (103.61 ± 45.48 ng / ml), folic acid (7.16 ± 5.88 ng / ml), vitamin B12 (160.59 ± 36.92 pg / ml) and transferrin (223.86 ± 44.14 mg / dl). Of the sample size of 44 primiparous people who attended the talks with caregivers, 35 (79.54%) breastfed their babies, while the other 44 primiparous women who attended alone, only seven (15.91%) established breastfeeding successfully.

**Conclusions:**

The implications for public health research are that the presence of a caregiver in health education programs modifies levels of prolactin, folic acid, vitamin B12, and transferrin, as well as increasing breastfeeding rates.

In the last two years, breastfeeding rates have experienced a notable decline worldwide. Only 46% of women breastfeed their children, the figure being much lower in primiparous women [1]. Currently, breastfeeding rates worldwide have been significantly reduced, the World Health Organization (WHO) [2], estimates that the percentage of infants exclusively breastfed up to six months is 41.0% [3]. Exclusive breastfeeding rates remain low in low-income countries (Nigeria, Colombia, Sierra Leone, Congo) as in the high income (United Arab Emirates, Germany, Sydney) [4].

For all age ranges, the highest percentages are presented in the countries of Sub-Saharan Africa and South Asia, where almost 70% of children continue to be breastfed at two years of age [5]. In Spain, according to data from the National Statistics Institute (Instituto Nacional de Estadística, INE), the exclusive breastfeeding rate at six months is 38.53% [6]. According to the latest studies, primiparous pregnant women do not breastfeed their children due to a lack of information on adequate nutrition during pregnancy to ensure the establishment of adequate lactation [6,7]. In 2019, the Spanish Pediatrics Association determined that 70.3% of pregnant women do not have an adequate diet, which may be the cause of the abandonment of breastfeeding [7]. Several studies show that babies who are not breastfed have a higher risk of getting sick [8]. This fact gives rise to an important public health problem that requires the need for study.

Breastfed milk is the ideal food for babies; its benefits for the health of mothers and babies are scientifically proven [8,9]. Furthermore, it constitutes an ancient habit intimately rooted in the family and cultural context. Therefore, the meticulous study of the caregiver is essential. Human milk is the food of choice in the first six months [9]. Thus, exclusive breastfeeding is recommended for up to six months; it should be maintained for up to two years or more [9,10]. The increase in the prevalence and duration of breastfeeding benefits the entire society [10]. The assessment of the nutritional status of a pregnant woman is essential for the establishment of successful breastfeeding [11]. The hormone responsible for the secretion of breast milk is called prolactin. It stimulates lactation in women during pregnancy and maintains milk supply during lactation. The prolactin test measures the level of this hormone in the blood. Normal prolactin levels are 80 to 400 nanogrammes per millilitre (ng / ml) [12]. An important nutritional parameter during pregnancy and lactation is folic acid (vitamin B9) [13,14], which contributes to the normal psychological function of the newborn [15]. Normal levels of folic acid in a healthy adult are 2.7 to 17.0 ng / ml. Another analytical parameter related to the nutritional status of pregnant women is vitamin B12 [16], involved in vital processes such as maintenance of the nervous system. Normal values for vitamin B12 in a healthy adult range between 200 and 900 picogrammes per millilitre (pg / ml) [17]. Additionally, iron contributes to the normal formation of red blood cells and haemoglobin [18,19]. Normal transferrin values of iron in a healthy adult range from 204 to 360 mgmes per decilitre (mg / dl) [20].

Pregnancy includes numerous physical as well as psychological and / or emotional changes that can have great repercussions on the health of a pregnant woman, the foetus, the family and social life [21].

The coronavirus disease (COVID-19) pandemic has brought forward the need for greater emotional and social support. It has forced an important change in the approach to health education: non-face-to-face consultation [22]. This has considerably impacted pregnant women [23] with changes in health promotion strategies [24], making them less active in health-disease processes [25].

All these changes lead women to experience fear in the face of ignorance of healthy habits. This fear is further accentuated in primiparous pregnant women; therefore, it is important that women have adequate support for the acquisition of healthy habits [26]. The caregiver provides support and care [27,28], that is, the main caregiver plays an active role in the patient's quality of life [29]. However, in the current health programmes aimed at pregnant women, the figure of the caregiver is not contemplated. Previously, the hypotheses during the COVID-19 pandemic on the non-initiation of breastfeeding have been analysed. Among them, the most incipient was support in care [30].

The research question that guided this study was: is the inclusion of the primiparous pregnant woman’s primary caregiver in health education programs in the context of the COVID-19 pandemic influence on the acquisition of knowledge about nutrition during pregnancy (a through pre-and post-educational intervention) and this is evidenced in an adequate nutritional status of the pregnant woman to establish a successful breastfeeding?

## METHODS

### Study design

A prospective (cohort), descriptive and inferential study was carried out measuring the variable and design with intervention, where exposed and unexposed individuals are selected, to a factor (pregnant / main caregivers’ inclusion or exclusion in health education) with pre and post intervention and measurement, and after delivery, the rate of breastfeeding was assessed.

### Description of random variables

Continuous quantitative variables were prolactin, folic acid, B12 vitamin, and transferrin levels. These parameters were measured before and after providing health education. Nominal qualitative variables were the inclusion of main caregiver in health education, taking food supplements, and breastfeeding rate.

### Setting

The study was conducted at the Portillo Health Center (Valladolid), in a rural health area in the central region of Spain. The study was conducted in between March 17, 2020, and October 20, 2021.

### Participants

The sample was obtained retrospectively by selecting all the medical records of primiparous pregnant women under the codification of the clinical process of normal pregnancy in the Medora@ computer registry [31,32]. The patients were divided into two samples with independent data, where the primiparous women and caregivers were divided into two groups: group A with 44 pregnant women and group B with the rest 44 pregnant women and caregiver. Half of the sample were randomly selected to establish exposed and unexposed individuals to a factor [33,34] (in this case, the inclusion of caregivers in health education) to assess the degree of influence of health education on the caregiver for correct nutritional provision of the pregnant woman, promotion of breastfeeding, and its rate. The intervention corresponded to the choice of the sample according to variables and the application of health education sessions with or without the inclusion of the main caregiver with subsequent analysis.

### Inclusion criteria

The inclusion criteria were: 1) primiparous women who belonged to the Portillo Health Center (Valladolid, Spain) with a diagnosis of normal pregnancy registered in the clinical history; 2) women treated at the Portillo Health Center during the period from December 2020 to October 2021; and 3) primary caregiver identified by the pregnant woman during the pregnancy and puerperium process, able to give informed consent.

### Exclusion criteria

The exclusion criteria were: 1) pregnant women with no identified primary caregiver or referral; 2) primiparous women with a serious clinical situation, complicated or pathological pregnancy; 3) primiparous women with a diagnosis of coronavirus infection during pregnancy, three months before becoming pregnant or during breastfeeding (to not obtain interference in the analytical parameters); and 4) primiparous women in contact isolation situation or symptoms compatible with COVID-19 infection without laboratory confirmation of infection.

### Sample size

The research population consisted of pregnant primiparous women (n = 88) from the health centre in a region of central Spain. Using the sample formula, the sample size required for this population, a non-homogeneous structure, is within the 95% confidence interval, with a sampling error of ±5% n = 89 (1. 96) 2 (0. 2) (0. 8) / (0. 05) 2 (89 – 1) + (1. 96) 2 (0. 2) (0. 8) = 65.

### Intervention description

**Figure 1:**
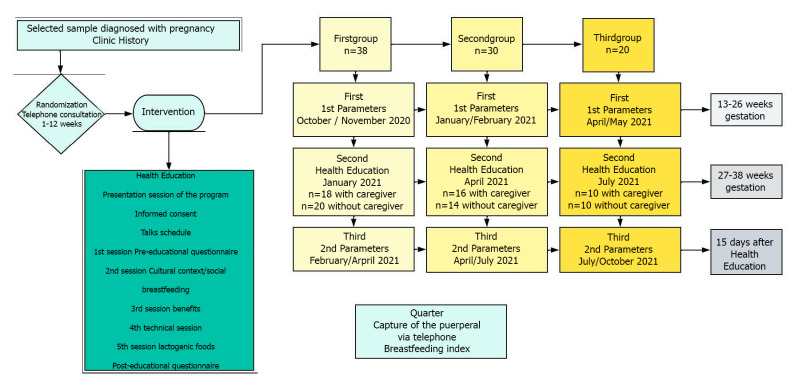
Recruitment and interventions into three groups.

The sample is divided into three groups with four previously planned stages of intervention (**Figure 1**). The study is structured in the following four interventions for each of the groups. 

First intervention: a session was held to present the project to the health team and request their collaboration. The midwives were contacted and asked for their collaboration to select the sample and contact the pregnant women. The main caregiver was recruited. Samples were collected after reviewing the records in nominal and numerals in clinical processes of pregnancy in the computerized medical history program (Medora@) [35] under due legal consent of the Primary Care Management (Valladolid, Spain), to access the Clinical History for research purposes. The pregnant woman and caregiver were contacted by telephone. They were interviewed and the project was explained. Once the women agreed to participate in the study after due informed consent, the first intervention was performed in the second trimester (13-26 weeks): blood parameters: prolactin, folic acid, B12 vitamin, and transferrin levels. 

Second intervention: next, a double health education intervention was carried out, established in two sample groups (27-38 weeks of pregnancy) previously defined, to carry out a comparison. Health education talks on breastfeeding were given to half of the sample, where the benefits for the mother, the baby and the family were explained [36] including technique and lactogenic foods. Whereas the other half of the sample received the same health education talks with the participation of their main caregiver during the last weeks of pregnancy. The level of knowledge of the two groups before and after the educational intervention was compared, through previously validated pre-educational and post-educational questionnaires [37], the purpose was to know the effectiveness of health education in terms of acquiring knowledge to carry out healthy eating habits during pregnancy with the aim of promoting breastfeeding, these questionnaires had two main ideas or contents to be developed in their different questions: knowledge of healthy foods to consume during pregnancy to increase milk production, lactogenic foods, and the importance of family support. All were followed up until the first postpartum visit, and then the breastfeeding index was assessed. For the correct collection of the blood sample (prolactin, folic acid, B12 vitamin, and transferrin levels), each participant was instructed regarding the ideal conditions to follow: do not exercise two hours before sample collection, be relaxed 30 minutes before, avoid stressful situations, avoid a diet rich in proteins and fats the day before the sample collection, fast for 8-10 hours, and do not take medications that can raise or lower the value [37,38]. Material resources: the extraction technique was performed by venipuncture in veins located in the antecubital area with a 21G butterfly nut with a BDVacutainer Safety Lok@ adapter, a 2.5cmx45cm latex venous compressor and a tube with separating gel (yellow cap) for prolactin, folic acid, B12 vitamin, and transferrin samples. This tube is without anticoagulant and contains serum separator gel and cellular elements, which keeps the serum stable for more than 48 hours and no obvious change in its biochemical characteristics and chemical compositions occurs [38]. For haematology and biochemical determinations, blood was taken in a reference hospital laboratory (University Clinical Hospital, Valladolid, Spain). For the study, the necessary material and human resources were counted, without interfering with the performance of other studies or tasks that nurses are usually entrusted with. The material resources used were Microsoft® PowerPoint® presentations with projected videos. Due to the current health situation of the COVID-19 pandemic, health education was carried out by virtually applying and respecting all health security measures. For the design of the health education talks, a group didactic program was carried out favouring the social participation of pregnant women [39]. An important aspect used in the intervention is the work in the cognitive, affective-attitudinal, and psychomotor spheres [40]. Oral, gestural, visual, and human support were emphasized, which served to transmit an idea, stimulate, motivate, and help change [41]. In addition, the effective transmission of messages was ensured to achieve a reaction, response, or impact [42]. For the educational intervention study, a schedule was previously planned. Virtual session to present the program: the study was carried out and the planning of the sessions (human and material resources) were explained. Health education talks: first session: preparation of the pre-educational questionnaire and staging of the statistical data / prevalence of breastfeeding at global, national, and regional levels; second session: presentation of the historical evolution. Cultural and / or social context. Third session: benefits of breastfeeding for the baby, mother, and family. Fourth session: breastfeeding technique and duration. Fifth session: adequate nutrition during pregnancy (lactogenic foods), and preparation of the post-educational questionnaire [43].

Third intervention: After the educational intervention and exposure of the sample population to the factor (health education), blood parameters were analysed in the third trimester of pregnancy (38-40 weeks) to compare the values once the health education has been imparted to pregnant women with or without their caregiver and assess the successful establishment of breastfeeding as the exclusive feeding for a baby.

Fourth intervention: postpartum women were recruited once they were discharged from hospital to assess the establishment of breastfeeding and interpretation of the results after the intervention (**Figure 1**).

### Data collection and measurements

Data analysis was performed using SPSS 20.0 software. To access the clinical history, the Medora@ computer program was used, and the following data were recorded: pregnancy diagnosis, health education activity with or without a caregiver, delivery date, puerperium diagnosis, and breastfeeding or not.

### Statistical analysis

To evaluate the data, frequency, percentage, mean, and standard deviation, ANOVA and χ^2^ analysis of variance were used. For the results, it was accepted as statistically significant *P* < 0.05. The identification of each patient was collected based on a medical record number to respect their confidentiality.

## RESULTS

The research population consisted of pregnant primiparous women (n = 88) with two groups for intervention: A (44 pregnant women with caregivers) and B (44 without caregivers).

### Association between the mean analytical values and the intervention carried out on the main caregiver in health education

The results obtained in the study show how the sample means of the analytical values of prolactin, folic acid, vitamin B12, and transferrin are modified with the presence of the caregiver of the primiparous pregnant woman before and after receiving health education talks (n = 44). At the time of data collection, the mean level of prolactin in primiparous women after attending caregiver-present health education (288.57 ± 107.46 ng / ml) was significantly higher than the prolactin levels of primiparous patients who attended without a caregiver. According to the data, primiparous women who attended health education with a caregiver had lower prolactin levels before receiving health education talks. This indicates the benefit of health education in the pregnant population and the importance of including their caregiver in these health education talks. The sample measures of folic acid, vitamin B12, and transferrin were homogeneously modified to the sample means of prolactin, being the highest levels in primiparous women after receiving health education in the presence of their caregiver as shown in **Table 1**.

**Table 1:** Association between the mean analytical values and the intervention carried out on the main caregiver in health education (n = 44 per intervention group and n = 88 for the overall sample)

	Caregiver presence (n = 44)	Average values pre-education	*P*-value	Average values post-education	*P*-value
**Prolactin (ng / ml)**	Yes	44.64 ± 11.84	0.001	288.57 ± 107.46	0.000
	No	83.03 ± 41.51		103.61 ± 45.48	
**Folic acid (ng / ml)**	Yes	5.26 ± 3.69	0.075	16.93 ± 4.09	0.000
	No	7.01 ± 5.07		7.16 ± 5.88	
**Vitamin B12 (pg / ml)**	Yes	225.14 ± 69.84	0.002	505.05 ± 213.97	0.000
	No	170.98 ± 32.93		160.59 ± 36.92	
**Transferrin (mg / dl)**	Yes	161.86 ± 38.96	0.001	296.82 ± 67.61	0.000
	No	218.45 ± 46.26		223.86 ± 44.14	

ng – nanogramme, ml – millilitre, pg –picogramme, mg – milligramme, dl – decilitre

### Association between breastfeeding rates and the intervention carried out on the main caregiver in health education

The increase in analytical values in the primiparous women with the presence of the caregiver also correlates with an increase in breastfeeding, that is, those women who attended the health education talks with their caregivers had significantly higher analytical values than the primiparous women who attended alone, and the former also had a higher rate of breastfeeding successfully. Women who breastfed their children and were accompanied to health education talks by their caregivers had prolactin levels of 295.76 ± 108.78. Of the sample size of 44 primiparous people who attended the talks with caregivers, 35 (79.54%) breastfed their babies, while the other 44 primiparous women who attended alone, only seven (15.91%) established breastfeeding successfully. Thus, it can be said that the presence of the caregiver enables greater breastfeeding, and, therefore, improvement of analytical values. If the variables breastfeeding and caregiver are compared for the analytical values before and after receiving health education, it is observed that the primiparous women, regardless of breastfeeding or not, have a caregiver score higher than those that do not have a caregiver for the different analytical values, in a statistically significant way (**Table 2**).

**Table 2:** Association between breastfeeding rates and the intervention carried out on the main caregiver in health education

Breastfeeding	Values analytical	Intervention educational	Presence of caregiver	SD	SEM
No	Prolactin (ng / ml)	Pre-education	No (n = 37)	83.03 ± 39.46	6.48657
			Yes (n = 9)	48.55 ± 7.11	2.36969
		Post-education	No (n = 37)	101.40 ± 35.30	5.80448
			Yes (n = 9)	260.57 ± 103.32	34.44106
	Folic acid (ng / ml)	Pre-education	No (n = 37)	7.20 ± 5.19	0.85322
			Yes (n = 9)	6.12 ± 2.28	0.76142
		Post-education	No (n = 37)	7.78 ± 6.01	0.98932
			Yes (n = 9)	14.37 ± 5.85	1.95144
	Vitamin B12 (pg / ml)	Pre-education	No (n = 37)	168.57 ± 32.32	5.31282
			Yes (n = 9)	198.44 ± 74.74	24.91380
		Post-education	No (n = 37)	162.19 ± 36.91	6.06767
			Yes (n = 9)	293.22 ± 118.10	39.36679
	Transferrin (mg / dl)	Pre-education	No (n = 37)	218.60 ± 46.77	7.68832
			Yes (n = 9)	163.11 ± 25.84	8.61434
		Post-education	No (n = 37)	224.76 ± 43.11	7.09
			Yes (n = 9)	276.66 ± 98.10	32.69684
Yes	Prolactin (ng / ml)	Pre-education	No (n = 7)	83.03 ± 54.84	20.72934
			Yes (n = 35)	43.63 ± 12.66	2.14
		Post-education	No (n = 7)	115.28 ± 84.58	31.96704
			Yes (n = 35)	295.76 ± 108.78	18.38704
	Folic acid (ng / ml)	Pre-education	No (n = 7)	5.98 ± 4.60	1.73836
			Yes (n = 35)	5.04 ± 3.98	0.67
		Post-education	No (n = 7)	3.90 ± 3.96	1.49379
			Yes (n = 35)	17.60 ± 3.30	0.55842
	Vitamin B12 (pg / ml)	Pre-education	No (n = 7)	183.71 ± 35.81	13.53429
			Yes (n = 35)	232.00 ± 67.95	11.48554
		Post-education	No (n = 7)	152.14 ± 38.69	14.62455
			Yes (n = 35)	559.51 ± 199.21	33.67348
	Transferrin (mg / dl)	Pre-education	No (n = 7)	217.71 ± 47.03	17.77735
			Yes (n = 35)	161.54 ± 41.98	7.09567
		Post-education	No (n = 7)	219.14 ± 52.73	19.93015
			Yes (n = 35)	302.00 ± 58.15	9.82947

SD – standard deviation, SEM – standard error of the mean, ng – nanogramme, ml – millilitre, pg – picogramme, mg – milligramme, dl – decilitre

### Association between breastfeeding rates, prolactin levels and intake of food supplements

Regarding the intake of food supplements, the sample mean of prolactin, folic acid, vitamin B12, and transferrin, does not detect a statistically significant increase (*P* > 0.05). That is, the women who did take supplements did not present increased values of the analytical parameters studied nor was there a significant increase in the rate of breastfeeding, as shown in **Table 3**. Pregnant women who take food supplements and breastfeed have lower prolactin levels (240.22 ± 119.63 ng / ml) than pregnant women who do not take food supplements and breastfeed (**Table 4**).

**Table 3:** Association between health education and intake of food supplements

	Food complements (n = 44)	Average values pre-education	*P*-value	Average values post-education	*P*-value
**Prolactin (ng / ml)**	Yes	64.98 ± 35.61	0.801	194.03 ± 117.9	0.850
	No	63.97 ± 36.98		199.18 ± 134.46	
**Folic acid (ng / ml)**	Yes	5.56 ± 4.33	0.140	11.53 ± 7.38	0.410
	No	6.98 ± 4.66		12.81 ± 6.49	
**Vitamin B12 (pg / ml)**	Yes	204.26 ± 59.94	0.241	361.20 ± 243.54	0.151
	No	188.65 ± 61.58		289.82 ± 206.20	
**Transferrin (mg / dl)**	Yes	182.50 ± 47.97	0.082	255.16 ± 66.6	0.382
	No	201.74 ± 54.3		268.17 ± 69.28	

ng – nanogramme, m – millilitre, pg – pictogramme, mg – milligramme, dl – decilitre

**Table 4:** Association between breastfeeding rates, prolactin, folic acid, vitamin B12 and transferrin levels and intake of food supplements

Breastfeeding	Values analytical	Intervention educational	Food complements	Average values
No	Prolactin (ng / ml)	Pre-education	No (n = 25)	76.99 ± 37.07
			Yes (n = 21)	75.44 ± 40.02
		Post-education	No (n = 25)	140.00 ± 92.13
			Yes (n = 21)	123.66 ± 73.11
	Folic acid (ng / ml)	Pre-education	No (n = 25)	7.20 ± 5.19
			Yes (n = 21)	6.12 ± 2.28
		Post-education	No (n = 25)	10.54 ± 6.24
			Yes (n = 21)	7.31 ± 6.46
	Vitamin B12 (pg / ml)	Pre-education	No (n = 25)	171.12 ± 39.52
			Yes (n = 21)	178.33 ± 50.33
		Post-education	No (n = 25)	189.28 ± 92.38
			Yes (n = 21)	186.09 ± 63.22
	Transferrin (mg / dl)	Pre-education	No (n = 25)	221.52 ± 49.00
			Yes (n = 21)	191.33 ± 43.78
		Post-education	No (n = 37)	252.44 ± 68.09
			Yes (n = 21)	214.04 ± 42.00
Yes	Prolactin (ng / ml)	Pre-education	No (n = 25)	34.98 ± 10.13
			Yes (n = 21)	54.96 ± 30.37
		Post-education	No (n = 25)	347.15 ± 107.75
			Yes (n = 21)	347.15 ± 107.75
	Folic acid (ng / ml)	Pre-education	No (n = 25)	6.38 ± 3.17
			Yes (n = 21)	4.82 ± 4.25
		Post-education	No (n = 25)	18.50 ± 2.28
			Yes (n = 21)	14.31 ± 6.66
	Vitamin B12 (pg / ml)	Pre-education	No (n = 25)	232.50 ± 84.68
			Yes (n = 21)	221.28 ± 60.31
		Post-education	No (n = 25)	541.20 ± 198.96
			Yes (n = 21)	476.12 ± 250.02
	Transferrin (mg / dl)	Pre-education	No (n = 25)	152.30 ± 30.59
			Yes (n = 21)	176.71 ± 50.36
		Post-education	No (n = 25)	307.50 ± 57.98
			Yes (n = 21)	282.15 ± 66.37

ng – nanogramme, m – millilitre, pg – pictogramme, mg – milligramme, dl – decilitre

## DISCUSSION

This study identifies how the inclusion of the caregiver in health education programs during the COVID-19 pandemic positively modifies the levels of prolactin, folic acid, vitamin B12, and transferrin in primiparous women, and is related to the establishment of successful breastfeeding. The analytical parameters studied in the primiparous pregnant woman were dependent on health education, including the caregiver. The only variable that modifies the analytical parameters is the presence of the caregiver, which confirms the need to implement a specific education program for the health of pregnant women, including the main caregiver in the clinical practice as soon as possible.

The adequate nutritional status of a pregnant woman is decisive for breastfeeding to be successfully established. According to the data obtained in this study, the participation of the caregiver in health education significantly influences the nutritional status of the primiparous woman. In a recent study [44], the following were found: the parallel increase in androgens, as the body mass index (BMI) in pregnant women increases, negatively influences the start and duration of breastfeeding, and in turn the start of lactogenesis II occurs late in women with altered feeding. It was, therefore, concluded that obese women were associated with significantly lower rates of initiation, duration, and exclusivity of breastfeeding. In our study, there was a statistical difference between transferrin and B12 vitamin parameters in pregnant women with and without a caregiver. The pregnant women who attended the health education talks with a caregiver had superior transferrin compared to values of those who attended without a caregiver; similar results were obtained for B12 vitamin levels.

The findings confirm the influence of social relations on the diet of pregnant women. Therefore, there is a significant difference between the rate of breastfeeding and the presence of a pregnant woman's caregiver in health education (*P* < 0.05) with social support being influential in promoting this health, habit for mother, child, family, and society.

Other research [45] has shown that obese women have reduced basal prolactin levels in the first 48 hours postpartum and reduced suckling-induced prolactin release from two to seven days postpartum, which may reduce the rate of milk synthesis during pregnancy in this period. Furthermore, it indicates that obesity during pregnancy (BMI > or = 30.0) is associated with a short duration of breastfeeding. Additionally, an increase of 0.7 kilogrammes (kg) of additional weight of the newborn during infancy should also be considered. In our study, the prolactin levels of pregnant women who attend adequate nutrition sessions for a correct establishment and duration of breastfeeding were higher than the levels before attending the sessions (43.63-12.66 ng / ml, n = 44).

In reference to the importance of caregivers in health education, an investigation carried out in controlled pregnant women in hospital outpatient clinics [46] concluded that educational support to mothers regarding breastfeeding, either before or after childbirth, improves the proportion of mothers who maintain breastfeeding at six months of life of the newborn. A significantly higher percentage of mothers with exclusive breastfeeding was found in the group that received prenatal education at six weeks 1.04 ± 2.90 and at three months 1.07 ± 3.48. In our study, the rate of breastfeeding in primiparous women with a caregiver after receiving health education was 79.54%; while only 15.91% primiparas women who attended without a caregiver, breastfed their children.

In our study sample, it was observed that the levels of prolactin, folic acid, vitamin B12, and transferrin were higher in pregnant women who attended health education with a caregiver than in pregnant women who attended alone. This is similar to the nutritional parameters of folic acid, vitamin B12, and transferrin, the values being dependent on the caregiver variable. A recent study [47] confirms how the social relations of the pregnant woman are statistically significant in the correct feeding of the woman and the success of breastfeeding. This study aimed to summarize the existing research on the possible causes of the reduction in the incidence, exclusivity, and duration of breastfeeding in obese women. As a result, they were found that obese women demonstrated reduced confidence in their ability to achieve their own breastfeeding goals (*P* < 0.0001), these women had fewer close friends and relatives with previous breastfeeding experience; they also had less social influence to breastfeed. In our study, the important role of the caregiver in the care of the primiparous pregnant woman in terms of her diet is objectified, which has repercussions on the increase in the rate of breastfeeding; therefore, the health intervention of the “caregiver” element is necessary to increase the rates of breastfeeding.

A study of nutritional supplementation with omega 3 acids for the breastfeeding mother raised the possibility of modifying breast milk through an intervention and the importance of the specific modified nutrient, in this case, docosahexaenoic acid (DHA), in child growth and development [48]. However, in our study, it was found that taking supplements does not improve analytical values or breastfeeding, pregnant women who breastfed and took supplements had prolactin values of 240.22 ± 119.63; while in those who breastfed and did not take supplements, prolactin remained at higher values 347.15 ± 107.75.

In our study, the sample of pregnant women showed significantly lower prolactin values and nutritional parameters (folic acid, vitamin B12, and transferrin) before attending the health education sessions. The levels increased after receiving correct information on lactogenic foods, suitable for establishing successful breastfeeding. The results obtained are in line with the study on the consumption of ultra-processed foods by pregnant women through an educational intervention [49], which argued that the intervention reduced percentages of energy between the first and second trimesters of pregnancy by 4.6 points (*P* = 0.015). This effect was not seen in the third trimester of pregnancy, this study concluded that the training of health professionals to promote healthy eating practices is a viable and sustainable alternative to reduce the consumption of ultra-processed foods. It is important to point out that according to the Health Promotion and Prevention Strategy of the National Health System (Spain), the specific objective is to promote healthy lifestyles, defining the results of Health Promotion as measured and based processes in health education, where health professionals must acquire skills [50]. Our study confirms the importance of health education in reference to the feeding of pregnant women to increase the rate of breastfeeding, but this health education is extended with an important and innovative element, the caregiver of the mother pregnant woman. Patients benefit through health education that helps to maintain health under normal conditions, develop their abilities to face daily situations and be able to contribute to their community; this can also lead to lower health and social costs. However, for this, it is necessary to contribute to a more efficient allocation of the structure and resources of primary care [51].

COVID-19 pandemic has undoubtedly brought with it greater affective, emotional, and social vulnerability for pregnant women, as well as health professionals who work in pregnancy care services. A recent study shows the impact on the well-being in the United Kingdom nursing and midwifery workforce during the first wave of the pandemic, a revised Impact of Events score ≥33 (probable posttraumatic stress disorder) was observed in 44.6%, 37.1%, and 29.3% of participants [52]. It has changed the modality of health education, without the presence of a pregnant-health professional, resulting in a greater influence of caregiver on pregnant woman. Hence the need to provide the caregiver with adequate knowledge of lactogenic foods for the promotion of breastfeeding. A qualitative study [53], where semi-structured interviews were conducted with 60 postpartum women after having their first child, they responded that breastfeeding was perceived negatively, and this may be due to the difference between the real problems encountered and the idealized expectations conveyed by prenatal information. This study concludes that greater efforts are required from health care professionals in the maternity unit and in the community to provide mothers with adequate health education. According to our study, the differentiating element accounts for the importance of the caregiver in promoting the health of the pregnant women.

Regarding the generalization of the results, this study advances the knowledge of the degree of influence of the caregiver on the healthy eating habits of the pregnant woman to establish successful breastfeeding. Therefore, the need to restructure the current education programs for health in pregnant women is confirmed, including the caregiver as an agent of active change. Healthcare professionals, as those responsible for health education, must consider the active role of caregivers in pregnant women, besides knowing how they perceive pregnancy care. Pregnancy is a stage of vulnerability in women; this period requires a more complex management, devoting extra time, and overload on the part of the caregiver. For this reason, we must provide comprehensive and integrated care to the caregivers [54]. A study on nursing students from three Spanish public universities whose objective was to understand their knowledge about breastfeeding concluded that curricula based on real health practices related to motherhood are useful for future professionals to acquire adequate strategies of knowledge about breastfeeding. This fact confirms the findings of our study, which indicate that a common and comprehensive Health Education Protocol should be established to promote breastfeeding. This program must include assistance to the caregiver of the pregnant woman, carried out by the health professionals in both urban and rural areas. A common clinical guide [55] for standardized action throughout the health system is needed, for which it is essential that the health policy knows this evidence, as a final objective in the search for the improvement of the sanitary quality. According to a study [56], the use of telemedicine in the prevention and treatment of infectious diseases is expanding, with evidence of the potential impact on optimizing clinical outcomes and improving access to care; it may be important to establish telemedicine to spread health education to the caregiver.

## CONCLUSIONS

Our analysis shows a strong association between the presence of the caregiver with the primiparous woman in the health education programs and analytical values of prolactin, folic acid, vitamin B12, and transferrin, as well as increasing the rates of breastfeeding, that is, the presence of the caregiver has important repercussions in the promotion of healthy habits (adequate nutrition of the pregnant woman) to establish successful breastfeeding in a mother. This clearly shows the importance of including the caregiver in health education programs. Our research has also highlighted a difference in care that a pregnant woman receives with or without a caregiver, how it affects the acquisition of healthy habits and their state of health.

Finally, it should be noted that this intervention provides important advances in the scientific community, field of Public Health, and Health Promotion, since it evidences the need to implement in health centers a Health Education program for pregnant women, including the caregiver, under the prism of comprehensive health strategies. In this sense, the role of the nurse as an agent of change is paramount. This study projects a solution to the problem of low rates of breastfeeding, improving the quality of life of women, children, and society, in addition to reducing the cost of medical care.
